# Directional Torsion Sensor Based on a Paired Helical-Type Multimode Fiber

**DOI:** 10.3390/s25072091

**Published:** 2025-03-27

**Authors:** Wenlei Yang, Ke Tian, Le Li

**Affiliations:** 1 College of Information and Intelligence Engineering, Zhejiang Wanli University, Ningbo 315100, China; yangwenlei@hrbeu.edu.cn; 2 College of Physics and Optoelectronic Engineering, Harbin Engineering University, Harbin 150001, China; ketian@hrbeu.edu.cn

**Keywords:** torsion sensor, multimode interference, directional discrimination, optical fiber sensor

## Abstract

A torsion sensor with directional discrimination based on a single-mode–twisted multimode–single-mode (STMS) fiber structure is demonstrated. The STMS fiber structure is fabricated by fusion-splicing a segment of multimode fiber (MMF) between two single-mode fibers (SMFs), where the MMF is simultaneously heated and twisted to form paired helical-type structures. The experimental results indicate that the resonance wavelength shifts towards shorter wavelengths as the twist rate increases in the clockwise direction, while an opposite shift occurs in the counterclockwise direction. Within a twist rate range of −5.2 rad/m~5.2 rad/m, the wavelength and transmission sensitivities are determined to be −1.38 nm/rad·m^−1^ and 3.12 dB/rad·m^−1^, respectively. Furthermore, the proposed twist sensor exhibits minimal temperature crosstalk of 0.0072 (rad/m)/°C, making it highly desirable for mitigating temperature-induced cross-sensitivity in torsion measurement applications.

## 1. Introduction

Torsion measurement using fiber optic sensors has been widely applied across various fields, including the precise motion control of humanoid robots, the high-precision demands of the automotive industry, and the critical domain of structural health monitoring. A variety of torsion sensors utilizing optical fibers have been developed and proposed in the literature, including long-period gratings (LPGs) [1,2,3], fiber Bragg gratings (FBGs) [4,5,6], titled FBGs (TFBGs) [7], interferometers [8,9,10], and microfiber resonators [11]. However, many existing torsion sensors lack the capability to simultaneously measure both the twist rate and the twist direction [8,11,12]. In applications such as smart structures and environment monitoring, torsion sensors with high sensitivity and the ability to distinguish torsional directions are highly desirable. Consequently, the development of high-sensitivity, directionally discriminative torsion sensors has become increasingly important [13,14,15].

Recently, several fiber-optic torsion sensors utilizing fiber gratings and interferometric techniques have been shown to effectively measure both the twist rate and torsion direction concurrently [16,17,18]. For example, Liu et al. proposed a torsion sensor based on LPG by fabricated clockwise/anticlockwise fiber sheet structures to determine torsion direction. Consequently, the torsion sensor featuring clockwise-revolving fiber sheets (dextrorotation grating) exhibits a remarkable torsion sensitivity of 0.175 nm/rad·m^−1^, whereas the LPG with counterclockwise-revolving fiber sheets (levorotation grating) demonstrates a notable torsion sensitivity of −0.153 nm/rad·m^−1^ [19]. He et al. proposed a bidirectional torsion sensor based on micro tapered long-period fiber grating with arc discharge technology, and the torsion sensitivity of the sensor is −0.287 nm/rad·m^−1^ [20]. J Jiang et al. experimentally fabricated chiral long-period fiber grating (CLPG) in twisted double-cladding fiber through CO_2_ laser heating, achieving an impressive torsion sensitivity enhancement of −0.4 nm/rad·m^−1^ [21]. Afterward, Lu et al. described LPG created through the combination of CO_2_ laser polishing and pre-twisting, achieving a maximum measured torsion sensitivity of −0.654 nm/rad·m^−1^ [22]. In addition to the LPG structures, various interference torsion sensors based on special fibers for directional torsion measurement were reported recently, such as a torsion sensor utilizing directional coupling in twisted photonic crystal fiber, demonstrating torsion sensitivities as high as ~0.203 and ~0.208 nm/rad·m^−1^ in the clockwise and counterclockwise rotations [23]. A torsion sensor employing a multimode fiber arrangement with an integrated seven-core structure has been constructed, demonstrating torsional sensitivity values of 1.837 dB/rad·m^−1^ for clockwise rotation and −1.456 dB/rad·m^−1^ for counterclockwise rotation [24]. Similarly, a fiber-optic torsion sensor based on a helical two-core fiber has been proposed and experimentally demonstrated as being capable of simultaneously measuring both the torsion angle and the torsion direction [25]. More recently, an interference torsion sensor used for noncircular end-face dumbbell fiber was reported; the torsion-sensing sensitivity can be enhanced by enhancing the internal stress of fiber and the measured torsion sensitivities were 2.13 nm/rad·m^−1^ for the forward direction and −1.57 nm/rad·m^−1^ for the reverse direction [26]. These torsion sensors can realize the simultaneous measurement of twist rate and torsion direction. Nevertheless, many torsion sensors display significant temperature sensitivity, potentially leading to temperature-induced crosstalk issues. Furthermore, compared with torsion sensors made using special optical fibers, multimode optical fibers, as a type of optical fiber that is cost-effective, versatile, and efficient [27,28,29], demonstrate significant advantages in the design and development of low-cost and high-performance optical sensors.

In this paper, we propose a Mach–Zehnder interferometer (MZI) based on a singlemode–twisted multimode–single-mode (STMS) fiber structure, designed for directional torsion sensing measurements. The STMS fiber structure consists of a pair of co-directional helical-type structures in a ~4 cm long MMF. Due to the co-directional helical-type structures, the proposed STMS sensor has a high torsion sensitivity and the ability to identify rotational direction. In addition, STMS sensors have the advantage of being able to reduce torsion–temperature crosstalk effects with temperature sensitivity as low as 10 pm/°C. The STMS-based twist sensor proposed in this investigation is cost-effective and easy to fabricate, offering promising potential for applications in high-sensitivity torsion monitoring.

## 2. Analysis of Sensing Principles

**Figure 1:**
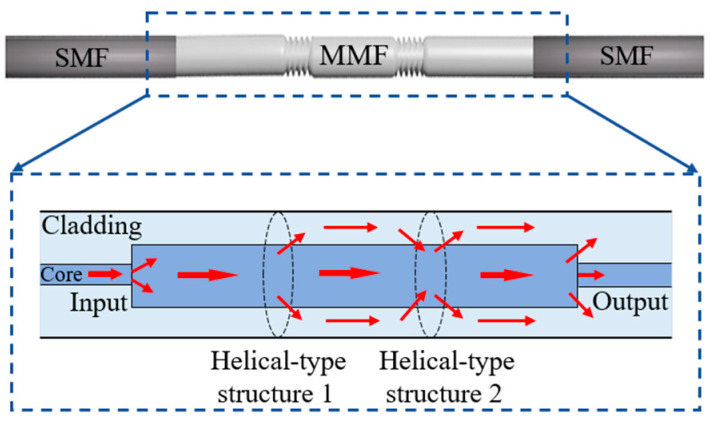
Schematic diagram of the STMS fiber structure and its light coupling mechanism within the two helical-type MMFs.

Figure 1 illustrates the STMS fiber structure, where the STMS fiber structure is configured by fusion splicing a short section of multimode fiber (MMF) with two helical-type structures between two single-mode fibers (SMFs). The MMF section incorporates two helical-type structures, which together form a Mach–Zehnder Interferometer (MZI) configuration. In the initial helical-type structure, higher-order modes from the multimode fiber (MMF) core propagate into the MMF cladding. These modes then propagate through the cladding region between the two helical structures before being coupled back into the fiber core at the second helical-type structure. Consequently, the introduction of two helical-type structures enhances the proportion of power in the evanescent field within the MMF cladding region. These configurations not only modify the shear stress but also adjust the local refractive indices within the multimode fiber (MMF). The multimode interference within the MMF section is substantially affected by the presence of both helical-type segments.

The extent and spatial scale of coupling generally differ from those in a straight configuration. When focusing primarily on the coupling within the degenerate mode manifold, *K* denotes the coupling rate per unit length, which can be assessed through integral calculations [30]:

(1) $$K_{\mu ,\nu }=\frac{\omega }{4}\int_{0}^{\infty }\int_{0}^{2\pi }rE_{\mu }^{*}\tilde{\varepsilon }E_{\nu }d\varphi dr$$

where $\tilde{\varepsilon }$ is the 3 × 3 matrix that encapsulates the perturbation term, which must be integrated into the dielectric tensor of the ideal fiber to yield the perturbed tensor. **E***_μ_* and **E***_ν_* are the complete electric fields of the modes *μ* and *ν* (the third component, denoted as *z*, corresponds to the fiber axis), and *r* and *φ* are the coordinates of a cylindrical reference frame.

Upon twisting a fiber, the shear stresses applied to the silica core, along with the associated elasto-optical phenomena, lead to a disturbance in the permittivity tensor, consequently altering the value of $\tilde{\varepsilon }$ [31]:

(2) $$\tilde{\varepsilon }=g\tau \varepsilon_{0}\bar{n}r\left[\begin{array}{ccc} 0 & 0 & -\sin\varphi \\ 0 & 0 & \cos\varphi \\ -\sin\varphi & \cos\varphi & 0 \end{array}\right]$$

where *g* signifies the elasto-optic coefficient, *τ* indicates the twist rate per unit length, and $\bar{n}$ embodies the average refractive index. As revealed by Equations (1) and (2), coupling between the linearly polarized modes LP*_n,p_* and LP*_m,q_* can only manifest if either *n* = *m* or the absolute difference |*n* − *m*| = 2; otherwise, integration over *φ* would ultimately produce a null result. In practice, alongside the elastic optical effects of torsion, the twisting action also induces geometric rotation in the fibers. Consequently, this rotational effect significantly influences the coupling of higher-order modes. Assuming the final coupling matrix is denoted as **K′**, let **X** represent the coupling matrix arising from the elasto-optic effects due to twist and **Y** denote the coupling matrix representing perturbations not induced by twist. When the fiber experiences twisting, inducing geometrical rotation, the coupling matrix **Y** evolves into **Y′** = **R**(*θ*)**YR***^T^*(*θ*), where *θ* denotes the angle of rotation and **R**(*θ*) = diag (**R***_i_*_1_(*θ*), **R***_i_*_2_(*θ*), …) embodies a block diagonal matrix [32]. The corresponding rotation matrices for the LP manifold are expressed as follows:

(3) $$R_{0}=\left[\begin{array}{cc} \cos\theta & -\sin\theta \\ \sin\theta & \cos\theta \end{array}\right],\;\;R_{n}=\left[\begin{array}{cc} R_{0} & 0\\ 0 & R_{0} \end{array}\right]\left[\begin{array}{cc} \mathrm{Icos}\;n\theta & -\mathrm{Isin}\;n\theta \\ \mathrm{Isin}\;n\theta & \mathrm{Icos}\;n\theta \end{array}\right]$$

where **R**_0_ represents the LP_0*,p*_ manifolds, and **R***_n_*(*θ*) denotes the LP*_n,p_* modes for *n* > 0. Based on the principle of superposition, the resulting coupling matrix is expressed as **K′** = **X** + **Y′**.

**Figure 2:**
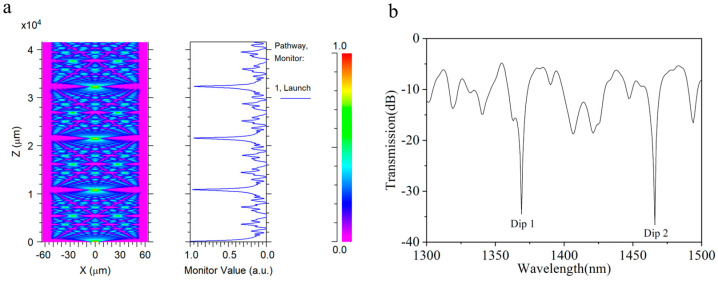
(**a**) Simulation of optical field distribution of STMS with two helical−type structures using BPM. (**b**) Simulated transmission spectrum of the STMS fiber structure.

Drawing upon the aforementioned theoretical analysis of mode coupling within the context of STMS fibers, the optical field distribution along the light propagation direction was simulated using the beam propagating method (BPM), as illustrated in Figure 2a. In the simulation, the mesh resolution in the X and Y directions was set to 0.1 μm, while the mesh resolution along the propagation direction Z was set to 1 μm. A perfectly matched layer (PML) boundary condition was employed in the model. Figure 2a demonstrates that the high-order modes of the multimode fiber (MMF) core emanate from the MMF core into the cladding at the initial helical structure, traverse the cladding region between the two helical structures, and are subsequently re-coupled back into the fiber core at the subsequent helical structure. Figure 2b shows the simulated transmission spectrum of the STMS fiber structure, featuring a 20 mm separation between the two helical segments, meticulously fabricated using a filament heater. It is readily apparent that distinct dips, engendered by mode interference, pervade the entire transmission spectrum, rendering these characteristics eminently suitable for sensing applications.

**Figure 3:**
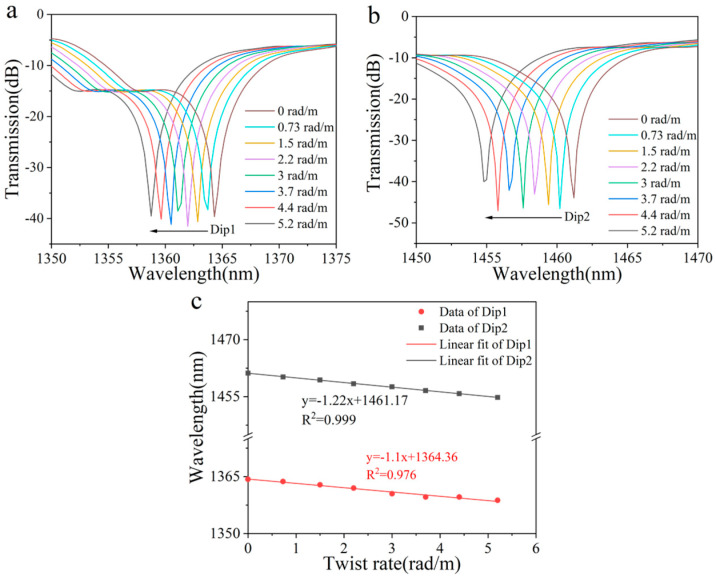
(**a**) Simulated transmission spectrum evolution of Dip 1 and (**b**) Dip 2 with increased clockwise twist. (**c**) Linear fitting results of the resonant wavelength shift versus twist rate.

Subsequently, a detailed simulation was conducted to investigate the sensor’s response under clockwise torsion. As shown in Figure 3a,b, the twist rate ranged from 0 to 5.2 rad/m, inducing a blue shift in the wavelengths of the two peaks. This blue shift became increasingly significant with an increase in the torsion rate. Furthermore, the wavelength variations in the two peaks exhibited a highly significant linear relationship with the changes in the twist rate, as depicted in Figure 3c. Following the fitting process, the torsion sensitivities of the two peaks were determined to be −1.1 nm/rad·m^−1^ and −1.22 nm/rad·m^−1^, respectively.

**Figure 4:**
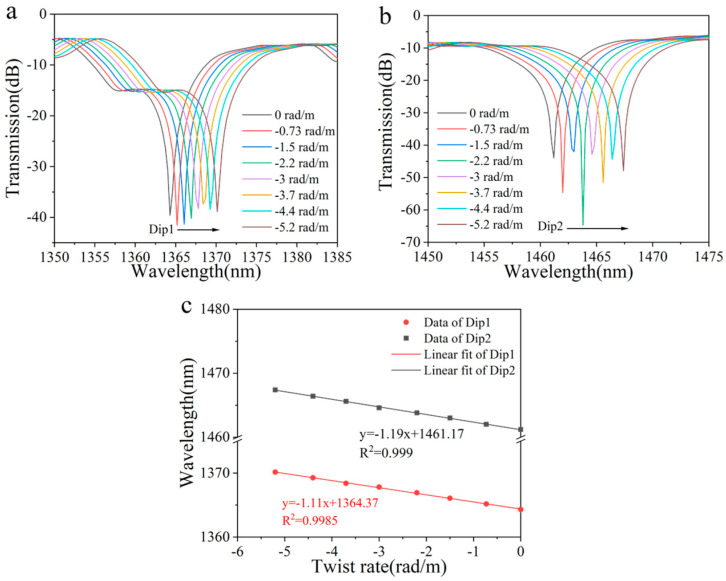
(**a**) Simulated transmission spectrum evolution of Dip 1 and (**b**) Dip 2 with increased counterclockwise twist. (**c**) Linear fitting results of the resonant wavelength shift versus twist rate.

Following this, a comprehensive set of simulations was conducted to examine the response characteristics of the sensor under counterclockwise twisting. As illustrated in Figure 4a,b, the twist rate was varied within the range of 0 to −5.2 rad/m, exhibiting behavior entirely opposite to that observed under clockwise twisting. This resulted in a red-shift of the wavelengths corresponding to the two peaks as the twist rate increased. Moreover, the wavelength changes in the two peaks demonstrated a strong linear correlation with the variations in twist rate, with correlation coefficients (R^2^) exceeding 0.99, as shown in Figure 4c. After performing the fitting process, the torsion sensitivities of the two peaks were calculated to be −1.11 nm/rad·m^−1^ and −1.19 nm/rad·m^−1^, respectively. Theoretical simulation results show that the sensor has the ability to distinguish the direction of torsion.

**Figure 5:**
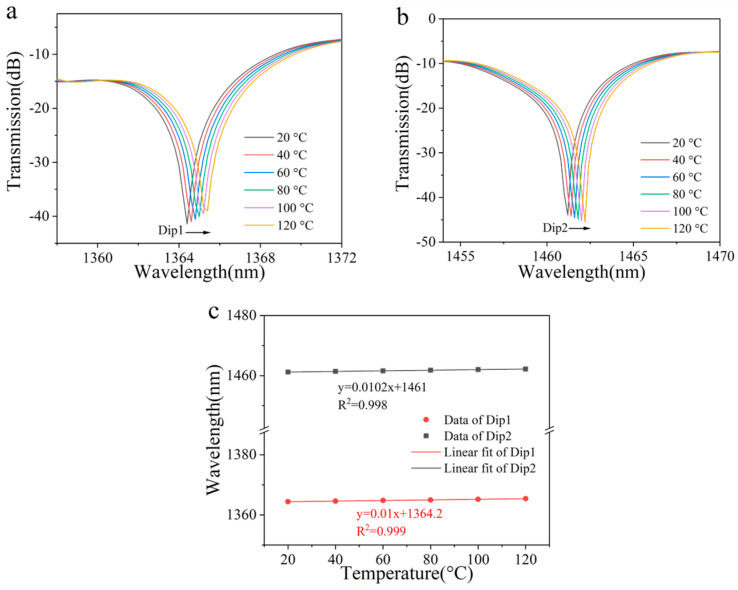
(**a**) Simulated transmission spectrum evolution of Dip 1 and (**b**) Dip 2 with increased temperature. (**c**) Linear fitting results of the resonant wavelength shift versus temperature.

Finally, we conducted a simulation and analysis of the sensor’s temperature response characteristics. The temperature range was set between 20 °C and 120 °C. As the temperature increased, the wavelengths of both peaks consistently shifted toward longer wavelengths, as shown in Figure 5a,b. Calculations revealed that the wavelengths of the two peaks exhibited a linear relationship with temperature changes, with respective temperature sensitivities of 10 pm/°C and 10.2 pm/°C. The simulation results are presented in Figure 5c. For single-mode or multi-core fiber interferometers, interference occurs between the core mode transmitted in the fiber core and the cladding mode transmitted in the cladding. Due to the different materials of the core and cladding, their thermal optical coefficients are also different, so the effective refractive index of the core mode and cladding mode varies greatly with temperature. In contrast, the interference in STMS proposed in this article is formed by different modes in multimode fibers, which are transmitted in the same medium (in the core of multimode fibers). Therefore, the difference in the effective refraction of different modes with temperature variation is much smaller than that in core-cladding mode interferometers, resulting in a decrease in temperature sensitivity. In addition, due to the addition of torsional structures on MMF, its torsion sensitivity has been significantly improved. The decrease in temperature sensitivity and the increase in torsion sensitivity ultimately result in minimal cross-sensitivity.

Based on the aforementioned theoretical analysis, the torsion–temperature cross-sensitivity (*C*) of the sensor can be calculated using the following formula:

(4) $$C=K_{Temperature}/K_{Torsion}$$

where *K_Temperature_* represents the temperature sensitivity of the STMS and *K_Torsion_* denotes the torsion sensitivity of the STMS. By substituting the temperature sensitivity value of 10.2 pm/°C and the torsion sensitivity value of 1.22 nm/rad·m^−1^ into Formula (4), we can obtain a torsion–temperature cross-sensitivity coefficient of 0.008 (rad/m)/°C.

## 3. Fabrication of STMS Sensor

**Figure 6:**
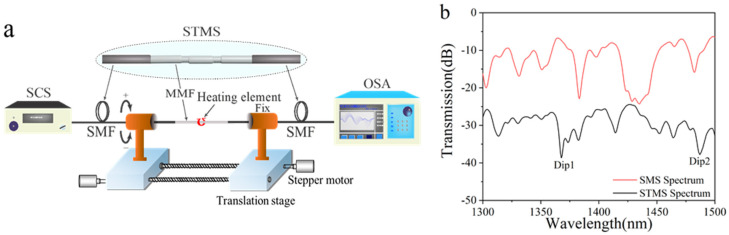
(**a**) Illustration of the setup for fabricating STMS fiber structures. (**b**) Measured transmission spectrum of the SMS and STMS fiber structures.

The schematic of the experimental setup is depicted in Figure 6a, with the fabrication processes outlined as follows. Initially, a standard SMS structure is prepared by splicing a 4 cm segment of step-index multimode fiber (AFS105/125Y) between two conventional single-mode fibers (SMFs). Subsequently, the fiber is secured, and a 1 mm region at the midpoint of the MMF is heated using a 50 W filament heater. During this process, the “+” and “−” symbols denote clockwise and counterclockwise twists, respectively. The initial helical structure of the MMF segment was continuously twisted two full revolutions in the clockwise direction at a constant rotational speed of 19°/s. The first helical-type structure was then fabricated. During the process of rotating, the fiber rotator was accurately controlled by the stepper motor. Thirdly, the fiber was moved using translation stages to produce the second helical-type structure with the identical twist direction and profiles as the first helical-type structure. The resulting separation between the paired helical-type structures was about 20 mm. Finally, the sample with an STMS fiber structure was fabricated. The fabricated sensor was characterized experimentally by interrogation using a super-continuum source (SCS) at the light source end, and an optical spectrum analyzer (OSA) with a spectral range spanning from 600 to 1700 nm was employed at the distal end. The measured transmission spectrums of the SMS and STMS are depicted in Figure 6b. As observed from Figure 6b, the transmission loss of the SMS sensor structure is −6.36 dB, while that of the STMS sensor is −24.43 dB. The transmission loss in the sensor arises due to the two twisted structures within the multimode fiber as well as the mode field mismatch between the single-mode and multimode fibers. The two prominent resonant dips are observed at wavelengths of 1367.8 nm (Dip 1) and 1487.2 nm (Dip 2). The maximum losses of both resonant dips are −38.6 dB of Dip 1 and −37.7 dB of Dip 2. The empirical findings agree with the theoretical predictions; however, minor discrepancies may arise owing to the inherent approximations in the physical parameters and torsional geometry within the theoretical simulations.

## 4. Experimental Measurements and Discussion

**Figure 7:**
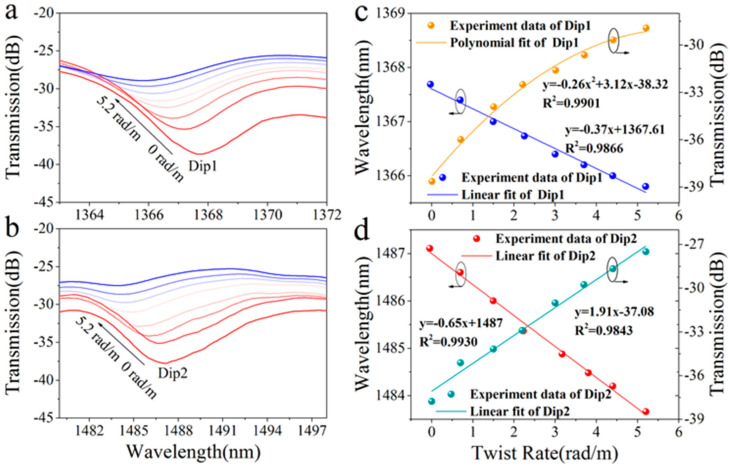
Spectral response of the STMS (**a**) at Dip 1 and (**b**) Dip 2 as the twisting rate was varied from 0 to 5.2 rad/m; resonant wavelength shift and depth of (**c**) Dip 1 and (**d**) Dip 2 as the twisting rate was varied from 0 to 5.2 rad/m.

The schematic diagram of the experimental setup for the STMS torsion sensor is illustrated in Figure 7a. One end of the STMS is securely fastened to a fiber holder, while the opposite end is connected to a precision rotator. The distance (*L*) between the fiber holder and the rotator measures approximately 236 mm, thereby enabling the application of a twisting effect when the rotator is actuated. The twisting rate was varied from 0 to 5.2 rad/m with 0.8 rad/m intervals, and the measured transmission spectra of the STMS fiber sample are shown in Figure 7a,b. Figure 7a,b show that when the STMS structure was twisted in a clockwise direction (co-directional with the two helical-types structures), the change in effective refractive index difference was negative by the co-directional twist, and the resonance dips exhibited a blue shift when the twisting rate increased. When the twisting rate was increased to 5.2 rad/m, the resonant wavelength of Dip 1 shifted from 1367.8 nm to 1366.0 nm, and Dip 2 shifted from 1487.2 nm to 1483.8 nm. Simultaneously, the maximum loss of Dip 1 changed from −38.7 dB to −28.9 dB and that of Dip 2 increased from −37.8 dB to −27.5 dB. The wavelength shifts of the resonant dips in response to the twist rate were measured and the corresponding numerically fitted curves are presented in Figure 7c,d. The resonant wavelength and resonant depth maximum twist sensitivities of Dip 1 are 0.37 nm/rad·m^−1^ and 3.12 dB/rad·m^−1^, and those of Dip 2 are −0.65 nm/rad·m^−1^ and 1.91 dB/rad·m^−1^.

**Figure 8:**
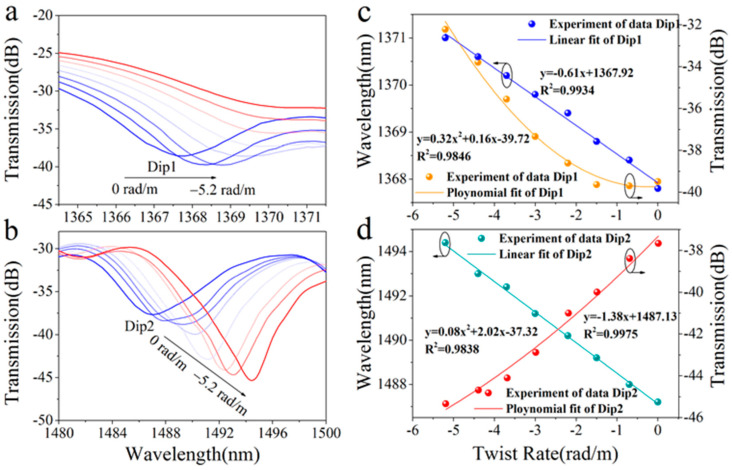
Spectral response of the STMS (**a**) at Dip 1 and (**b**) Dip 2 as the twisting rate was varied from 0 to −5.2 rad/m, resonant wavelength shifts and depths of (**c**) Dip 1 and (**d**) Dip 2 as the twisting rate was varied from 0 to −5.2 rad/m.

The twisting rate was varied from 0 to −5.2 rad/m with an interval of −0.8 rad/m, and the transmission spectra of the STMS fiber sample were measured and are shown in Figure 8a,b. A contra-directional twist was applied, resulting in a positive change in the effective refractive index difference, as clearly illustrated in Figure 8a,b; this causes the resonance dips to red shift, which increases as the twist rate decreases (becomes more negative). When the twisting rate was varied from 0 to −5.2 rad/m, the resonant wavelength of Dip 1 shifted from 1367.8 nm to 1371.0 nm, and Dip 2 shifted from 1487.2 nm to 1494.4 nm. Simultaneously, the loss of Dip 1 changed from −38.7 dB to −32.2 dB and that of Dip 2 changed from −37.8 dB to −45.3 dB. The calculated and measured wavelength shifts of resonant depths according to the twisting rate are shown in Figure 8c,d. The resonant wavelength and resonant depth maximum twist sensitivities of Dip 1 are −0.61 nm/rad·m^−1^ and 0.16 dB/rad·m^−1^, and those of Dip 2 are −1.38 nm/rad·m^−1^ and 2.02 dB/rad·m^−1^, respectively.

**Figure 9:**
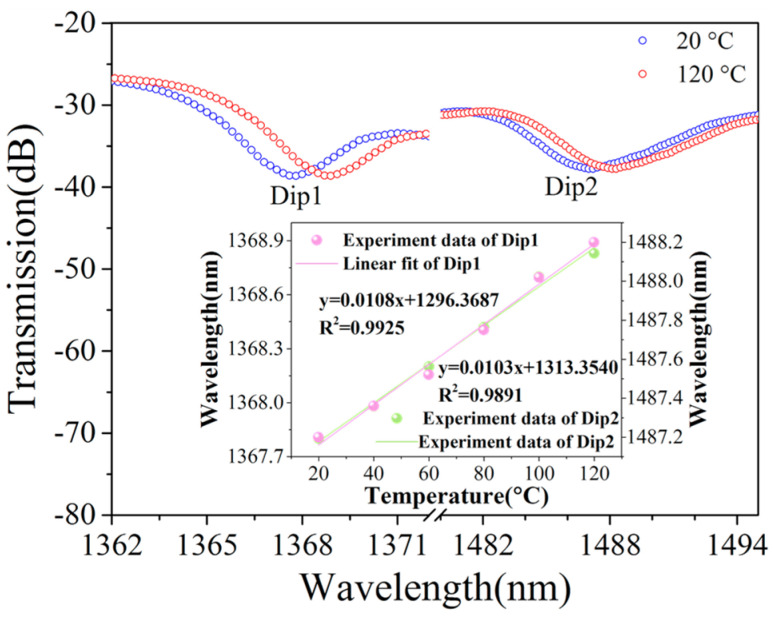
Measured transmission spectrum evolution of the STMS with increased temperature. Inset: linear fitting results of the resonant wavelength shift versus temperature.

The STMS sample was positioned within a temperature-controlled chamber to assess its thermal dependence. The transmission spectra of the STMS structure, as the temperature ranged from 20 °C to 120 °C, are illustrated in Figure 9. As the ambient temperature escalated from 20 °C to 120 °C, the measured wavelength shifts and resonant depth variation of two dips were only 1 nm and less than 0.1 dB, respectively. To ensure minimal influence from temperature fluctuations and achieve highly accurate twisting rate measurements, the environmental temperature was meticulously maintained at a stable level. Consequently, within a narrow temperature range, the twist sensor can be regarded as effectively temperature-insensitive. As shown in the inset of Figure 9, the temperature sensitivity of the STMS was calculated to be 10 pm/°C within the range of 20 to 120 °C.

**Figure 10:**
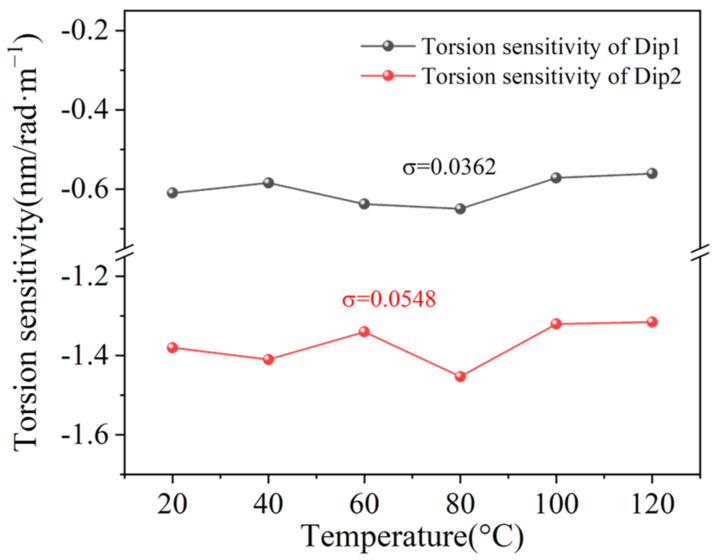
The torsion sensitivity responses with varied ambient temperature.

Subsequently, we evaluated the performance of our sensor in terms of availability across different temperatures. The relationships between torsion sensitivity and temperature are illustrated in Figure 10. According to the measured results, the standard deviations σ of the torsion sensitivities of two dips are 0.0362 and 0.0548, respectively. This tiny value of the standard deviation σ indicates that our sensor exhibits a stable response to torsion with a varied ambient temperature.

**Figure 11:**
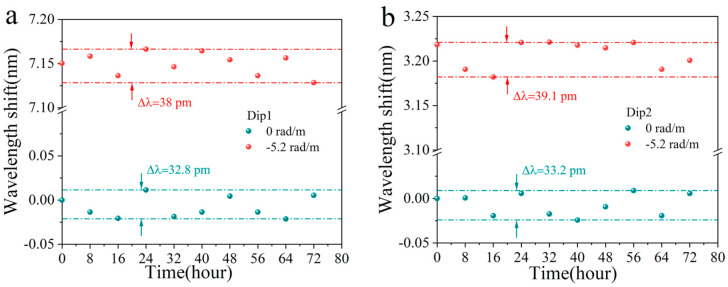
(**a**) Dip 1 and (**b**) Dip 2 stability verification results of the STMS.

Finally, we verify the stability of the sensor. The sensor was positioned at states of 0 rad/m and −5.2 rad/m respectively, and the relative wavelength shift of the two dips was meticulously recorded every 8 h. Data from ten experiments in each group are presented in Figure 11a,b below. The minor fluctuations in wavelength can be attributed to the interference caused by ambient environmental noise. In these two experiments, the maximum wavelength shifts were measured at 34.1 pm and 38.2 pm respectively, which remain significantly lower than the impact induced by a torsion change of 0.063 rad/m.

Table 1 compares the linearity, dynamic range, torsion sensitivity, temperature sensitivity, and temperature crosstalk of different types of torsion sensors. Compared with the characteristics of several sensors previously proposed, the STMS structure proposed in this paper exhibits superior torsion sensitivity and significantly reduced temperature crosstalk. Moreover, our manufacturing method is both simple and highly flexible, requiring neither expensive equipment nor specialized optical fibers.

## 5. Conclusions

In summary, a torsion sensor with directional discrimination has been experimentally demonstrated utilizing two identical helical structures within a multimode fiber. Upon rotating the sensor in a clockwise direction, the spectral wavelength experiences a blue shift. In contrast, a counterclockwise rotation of the sensor results in a red shift of the spectral wavelength. The direction of torsion can be accurately determined by observing variations in the spectral wavelength. For a twisting rate ranging from 0 rad/m to 5.2 rad/m, the maximum torsion sensitivities are −0.65 nm/rad·m^−1^ and 3.12 dB/rad·m^−1^, respectively. Conversely, for a twisting rate from −5.2 rad/m to 0 rad/m, the maximum torsion sensitivities reach −1.38 nm/rad·m^−1^ and 2.02 dB/rad·m^−1^, respectively. Compared to previously reported twist sensors, the proposed device offers advantages such as high torsion sensitivity, low cost, and ease of fabrication. Additionally, the sensor structure exhibits relatively low-temperature crosstalk, and it may have potential application prospects in the field of civil engineering.

## Figures and Tables

**Table 1:** The performance comparison of fiber-optic torsion sensors. In the table, “—” indicates “Not Given”.

Sensor Structure/Type	Torsion Sensitivity	Direction Discrimination	Linearity/ R-Square	Dynamic Range	Temperature Sensitivity	Temperature Crosstalk
MSM fiber structure [7]	−0.361 nm/rad·m^−1^	No	Good linearity/0.999	0~60 rad·m^−1^	1.792 nm/°C	4.96 (rad/m)/°C
Twisted MCF [33]	0.118 nm/rad·m^−1^	Yes	Good linearity/0.993	(−19.943~15.669) rad·m^−1^	101 pm/°C	0.86 (rad/m)/°C
Twisted PCF [23]	0.208 nm/rad·m^−1^	Yes	Good linearity/0.99	−76~76 rad·m^−1^	—	—
Helical two-core fiber [25]	0.242 nm/rad·m^−1^ and −0.04 dB/°	Yes	Non-linear/0.999	−60°~60°	32 pm/°C	0.13 (rad/m)/°C
Pre-twist LPFG [22]	−0.654 nm/rad·m^−1^	Yes	Good linearity/—	−12.6~12.6 rad·m^−1^	66.8 pm/°C	0.1 (rad/m)/°C
MMF–SCF–MMF [24]	1.837 dB/rad·m^−1^	Yes	Good linearity/—	0~3.5 rad·m^−1^	70 pm/°C and −0.296 dB/°C	0.16 (rad/m)/°C
Twisted MMF (this work)	−1.38 nm/rad·m^−1^	Yes	Good linearity/0.998	−5.2~5.2 rad·m^−1^	10 pm/°C	0.0072 (rad/m)/°C

## Data Availability

The data will be available upon request.
